# Awareness of Chronic Kidney Disease, Medication, and Laboratory Investigation among Nephrology and Urology Patients of Quetta, Pakistan

**DOI:** 10.3390/ijerph19095015

**Published:** 2022-04-20

**Authors:** Jahanzaib Ahmed, Saira Azhar, Noman ul Haq, Sajjad Hussain, Anette Stájer, Edit Urbán, Márió Gajdács, Shazia Jamshed

**Affiliations:** 1 College of Pharmacy, University of Sargodha, Sargodha 40100, Pakistan; miyanjahanzaib@gmail.com (J.A.); sajjadh44551@gmail.com (S.H.); 2 Faculty of Pharmacy and Health Sciences, University of Balochistan, Quetta 87300, Pakistan; noman.pharm@uob.edu.pk; 3 Department of Periodontology, Faculty of Dentistry, University of Szeged, 6720 Szeged, Hungary; stajer.anette@stoma.szote.u-szeged.hu; 4 Department of Medical Microbiology and Immunology, University of Pécs Medical School, Szigeti út 12., 7624 Pécs, Hungary; urban.edit@pte.hu; 5 Department of Oral Biology and Experimental Dental Research, Faculty of Dentistry, University of Szeged, 6720 Szeged, Hungary; 6 Department of Clinical Pharmacy and Practice, Faculty of Pharmacy, Universiti Sultan Zainal Abidin, Kuala Terengganu 21300, Malaysia; shaziajamshed@unisza.edu.my; 7 Qualitative Research-Methodological Application in Health Sciences Research Group, Kulliyyah of Pharmacy, International Islamic University Malaysia, Kuantan 25200, Malaysia

**Keywords:** awareness, knowledge, CKD, nephrology, urology, laboratory, medications

## Abstract

Patients’ awareness is critical in medical care, as it can serve as an input into the adjustment of interventions. The aim of study was to explore the level of awareness regarding chronic kidney disease (CKD), its medications, and laboratory investigations among nephrology and urology patients of Quetta. The cross-sectional study was used by adopting and culturally modifying a questionnaire. By convenient sampling technique, a total of 500 questionnaires were self-administered to inpatients, outpatients, and dialysis patients, and 468 responses (response rate 93.6%) were analyzed. Descriptive statistics, inferential statistics, and reliability analysis were performed on SPSS v25. A majority, 50.3% (*n* = 235), was unaware of symptoms that will develop due to worsening of disease, while 56.2% (*n* = 263) were unaware of what aggravates their kidney function. Almost half of the affected individuals, 47.4% (*n* = 222), have no understanding about the long-term prognosis of the disease. The majority of the respondents, 51.5% (*n* = 248), do not know about the names and usage of medications, and 62.4% (*n* = 292) were unaware of the medicines that may impair kidney function; more than half, 66.7% (*n* = 312), were unaware about the necessary laboratory investigations. A strong association between awareness and patient education level was found (*p* < 0.001). Awareness regarding disease condition, medications, and laboratory investigations of CKD among nephrology and urology patients of Quetta was found out to be low, which needs immediate educational intervention.

## 1. Introduction

Kidney damage or decreased renal function for three months or more is clinically considered as chronic kidney disease (CKD) [1]. In patients with CKD, the GFR (glomerular filtration rate) decreases, and/or there are urinary or structural problems in the renal system [2]. It is a progressive condition characterized by a decrease in kidney function of lower than 60 mL/min/1.7 m^2^. CKD is a major irreversible, gradual impairment in kidney function in which the body’s ability to maintain metabolic fluid and electrolyte balance fails. Renal function regulates blood composition and volume as well removes metabolic wastes by urination, which helps to maintain bodily acid/base balance [3]. In such cases, electrolyte imbalance may necessitate dialysis [3]. Several countries have listed CKD as one of the top five causes of mortality in 2015, according to the Global Burden of Disease report [4]. In Pakistan, prevalence of it is reported to be in between 12.5% to 31.2% [5]. CKD is becoming a considerable issue, as the incidence and prevalence of end-stage kidney disease (ESKD) have steadily increased over the last three decades [6].

The prevention of CKD is less costly as compared to its treatment and leading comorbidities [7,8]. CKD is a major public health concern that affects people all over the world; it has been identified as a serious public health hazard. Projections indicate that between 4.902 and 7.083 million people will have end-stage kidney disease and require renal replacement treatment around the world by 2050 [9]. CKD has a direct impact on the worldwide burden of morbidity and mortality because of its effect on cardiovascular risk and end-stage kidney disease [9].

Continuous therapeutic interventions and healthy lifestyle measures, such as appropriate nutrition, medications, and physical activity, are required for the well-being of patients affected by CKD [10,11]. To determine whether a treatment was effective, healthcare professionals (HCPs) and patients have traditionally relied on laboratory tests or subjective changes in the status of the patient’s condition. “Soft” metrics are those that focus on qualitative objective measurement. “Hard” metrics employ subjective data and measurable data to evaluate a patient’s concern. On the other hand, the emotional well-being, level of comfort, quality of life (QoL), and knowledge and awareness of patients are frequently overlooked when compiling these “hard” metrics even though these are significant factors affecting therapeutic success [12]. As a result, if precision in healthcare is to be applied, it is necessary to thoroughly examine each patient’s unique characteristics as well as ensure that the afflicted patients are aware of their own medical situation [13].

In patients with appropriate awareness of their health condition, the attitude and practices would change as a result of receiving new knowledge/information about their illness [14]. Patients’ awareness is critical in successful healthcare, as it is the foundation of the Knowledge–Attitude–Practice (KAP) Model [15]. As a result, it is imperative to accurately assess the patient’s awareness, as it can serve as an input for the planning and adjustment of interventions [16]. Everybody understands that long-term cooperation and shared decision making (SDM) between patients and HCPs is essentially required for the knowledge, awareness, and practice transition to be successful [17]. Age, ethnicity, being part of a minority group, education level, and socioeconomic status have all been linked to late referrals to a nephrologist for CKD [18]. Overall, it has been described that late referrals were linked to worse clinical outcomes and decreased QoL. As a result, low CKD knowledge and poor CKD awareness could have an impact on not only primary and secondary illness prevention but also on CKD progression and outcomes [18].

While much has been written about the success of screening programs in raising awareness of CKD both regionally and worldwide, there are few data pertaining to the general public’s real knowledge of the disease [18]. There has been quite a number of studies published regarding the knowledge and awareness of the general public on chronic diseases, such as hypertension [19], osteoporosis [20], human immunodeficiency virus (HIV) infection [21], polycystic ovary syndrome (PCOS) [22], and hepatitis virus B (HBV) infection [23], conducted in the Quetta valley, which is located in Balochistan. The Quetta valley is the largest population center in Balochistan province in western Pakistan [24]. It has a population of 1,001,205 individuals, according to the census of 2017 [25]. The country continues to suffer with inadequate health literacy, which leads to late disease presentation, poor treatment adherence, and a lack of knowledge about wellness and disease prevention. With poor healthcare facilities and low literacy levels in a country plagued by diseases from both the developing and developed worlds, enhancing healthcare literacy might have a significant impact on the health and well-being of our people [26]. CKD is a worldwide condition that has an impact on patients’ socioeconomic lives; however, little is known about the knowledge and awareness of the general population and the affected patients regarding CKD in Quetta.

Identifying knowledge gaps in patients affected by this condition regionally would be helpful to develop and improve patient education endeavors and managing chronic renal diseases; nevertheless, information regarding patient awareness on CKD is lacking in this region. The aim of the present study was to assess the level of CKD knowledge among specialty care patients in urology and nephrology in Quetta, including the exploration of the level of awareness regarding the condition, its medications, dietary risks, and laboratory investigations. To the best of our knowledge, this is the first cross-sectional study among nephrology and urology patients of Quetta about CKD illness awareness.

## 2. Materials and Methods

### 2.1. Study Design, Study Setting, Duration

The present quantitative, exploratory, cross-sectional, questionnaire-based study was performed at the Balochistan Institute of Nephrology and Urology Quetta (BINUQ), a public–private sector hospital and institution, which is the major specialized care hospital of nephrology and urology in the valley. The Balochistan Institute of Nephrology and Urology in Quetta is a state-of-the-art facility. BINUQ has successfully completed over 38 transplants and 2,950,000 dialysis sessions, serving 115 dialysis patients per day and treating 300 OPD patients per day [27]. The duration of the study ranged from March to December 2021, including the literature review, questionnaire development, questionnaire translation and validation, data collection, and manuscript writing. Data collection for the study was performed between August and September 2021 at BINUQ.

### 2.2. Study Population, Sample Size Determination, Sampling Technique

The research study was conducted on inpatients (including dialysis patients) and outpatients attending BINUQ. Patients with CKD, visiting for dialysis or for routine checkup, attending out-patient department (OPD), or admitted to wards were approached to participate in the study. The mean number of patients admitted to Balochistan Institute of Nephrology and Urology Quetta per a month were 104.0 ± 14.42 and 63.0 ± 30.32 at the nephrology and urology wards, respectively; the mean number of patients visiting the outpatient department were 4918.0 ± 1676.52; and the mean number of dialysis patients per month was 3216.0 ± 203.8 in 2019. Among these patients, only those patients who were willing to participate and to fill out the questionnaire and patients who could understand English and Urdu were included.

The data were collected through a convenient sampling technique. As the study was time-bound, the cross-sectional study design is best-suited for examining generally persistent conditions in a limited time frame. The required sample size was calculated using the Raosoft^TM^ [28] sample size calculator by taking a total average population of patients available per month in the hospital, which was approximately ~8400; with a margin of error of 5%, a confidence level of 95%, and response distribution as 50%, the required sample size was determined as *n* = 368. An additional factor of 20% for lost and incomplete responses was also added. Thus, a total 500 of questionnaires were administered to inpatients, outpatients, and dialysis patients randomly to collect the data; however, due to the short sampling time, following ethical considerations and taking into consideration the inclusion criteria and the refining of the data, *n* = 468 respondents were selected who willingly participated in this research study, which corresponds to a response rate of 93.6%.

### 2.3. Study Instrument, Translation, Reliability Analysis

A quantitative questionnaire consisting of questions about demographic information (age, gender, education level, and type of patient), clinical profile of the patient (duration of the disease, co-morbidities, lifestyle factors, and CKD stage), and questions related to the knowledge and awareness of CKD medications and laboratory tests was used. Prior to the study, a thorough literature analysis was conducted to establish the relevant questions and the study’s methodological problems. Lastly, the questionnaire used referred to an existing study [16,29] to assess the level of awareness among the patients; a modified questionnaire, following cross-cultural adaptation and validation, was used for data collection. The questionnaire was translated by standard instrument back-translation process [30,31], and after a consensus by all authors, the final version was developed. Firstly, the pre-validated English language version [16] was translated into the Urdu language, and afterwards, the Urdu version was back translated into the English version, and the two versions in the original language were compared to remove any obscurity from the finalized version, taking into account the cultural and linguistic aspects of the target population [32].

The finalized questionnaire (Supplementary Material S1) consists of three portions i.e., Demographic characteristics (age, gender, education level, and type of patient), Patient’s profile (duration of the disease, co-morbidities, lifestyle factors, and CKD stage) and 18-items specific for CKD. Item numbering is in the sequential order of the subsequent tables. Items 1–5 were regarding to awareness of CKD conditions, items 6–9 were regarding awareness of CKD medications, items 10–13 were regarding awareness of risk factors associated with the condition, items 14–15 were regarding awareness of laboratory investigations, and items 16–18 were associated with the awareness of management of the CKD. In these questions, three options for the answers were provided, i.e., “Yes”, “No”, and “Don’t Know”. During data analysis, the “Don’t Know” responses were merged with “No” in the results, as both are characteristic for the lack of awareness for the disease. The questionnaires were self-administered under supervision. There are people among the respondents who had no formal education, so the contents were explained and filled out accordingly in a few cases to avoid impact and ambiguity in data.

The internal consistency and reliability of the instrument was assessed using Cronbach’s alpha (Table 1); based on the value obtained (ά = 0.851), it corresponds to excellent reliability in questionnaire-based research.

### 2.4. Ethical Considerations

The study was approved by the Institutional Advanced Studies and Research Board, University of Sargodha, Punjab, Pakistan (Ref No. SU/Acad/1723; 22 November 2021). The research committee of the hospital provided us with the joining letter to start our data collection. Before data collection, the nature of the study and its aims and objectives were explained to the potential participants. Informed written consent was also collected from the participant before giving out the questionnaire, in which it was stated that their participation in the study was totally anonymous and voluntary, the information provided would be kept confidential, and they could skip any question that they did not want to answer. Consent was acquired from the parent/caregiver in the case of participants under 18 years of age. The written permission letter was approved by the Chief Executive Officer (CEO) of BINUQ to conduct this research.

### 2.5. Statistical Analysis

Demographic characteristics, including age, gender, education level, patient type, and clinical profile of patients, were tabulated, with descriptive statistics (mean ± standard deviation, frequencies, and percentages) provided for all items. Reliability analysis, normality test, and inferential statistics (chi-square tests) with variables were reported, and tests were performed using IBM SPSS^®^ for Windows (Chicago, IL, USA) v.25.0. *p*-Values < 0.05 were considered statistically significant.

## 3. Results

### 3.1. Demographic Characteristics

A summary on the demographic characteristics of participants are presented in Table 2. Among the respondents, mean age (±SD) was 30.16 ± 11.62 years, with the majority of respondents being males 74.1% (*n* = 347). A total of 52.6% (*n* = 246) of participants were outpatients, 45.1% (*n* = 211) were inpatient, and 2.4% (*n* = 11) were dialysis patients, and the majority of them were graduates 29.7% (*n* = 139).

### 3.2. Clinical Profile of Patients

The clinical profiling of participants related to their CKD condition is tabulated in Table 3. Among the respondents, almost three-quarters 73.9% (*n* = 346) were suffering from CKD for a duration of 1 to 3 years. Respondents included smokers (43.1%, *n* = 147) and 56.9% (*n* = 194) who exercised at least 30 min per day. The majority of participants 75.6% (*n* = 354) were at the first stage of CKD (i.e., having a glomerular filtration rate (GFR) of 90 mL/min/1.73 m^2^ or higher). In addition to CKD, many respondents had other co-morbidities as well, including hypertension (21%, *n* = 129), other cardiovascular diseases (including abnormal heart rhythms or arrhythmias, aortic diseases, congenital heart diseases, coronary artery disease, deep vein thrombosis and pulmonary embolism, history of a heart attack, and cardiomyopathy) (10.4%, *n* = 64), and diabetes (8.8%, *n* = 54).

### 3.3. Awareness Regarding CKD

As a principal part of this study, awareness of participants regarding their disease condition was assessed, with the responses of the participants presented in Table 4. Participants were asked if they know how to control their blood pressure, with the majority of the respondents (60.7%, *n* = 284) having a positive response, while 39.4% (*n* = 184) responded either negatively or indicating that they did not know about it. Two-thirds of the respondents (65.4%, *n* = 306) were aware that a person can lead a normal life with one kidney. More than half of the participants (50.3%, *n* = 235) were unaware of symptoms that develop due to worsening of disease, while (56.2%, *n* = 263) responded with either “No” or “Don’t know” about what aggravates their kidney function. Almost half of the affected individuals (47.4%, *n* = 222) have no understanding about the long-term prognosis of the disease.

Majority of the respondents 50.5% (*n* = 248) responded with “No” and/or “Don’t know” about knowing about the brand names and usage of their medications, while 55.5% (*n* = 260) responded with “No” and/or “Don’t know” about knowing the roles of their taken medicines. A total of 62.4% (*n* = 292) were unaware of the medicines that may impair their kidney function. More than half of the respondents, 57.5% (*n* = 269), responded with either “No” or “Don’t know” in relation to knowing about herbal medicines that could be effective in CKD. Further, 51.9% (*n* = 243) were aware about unhealthy diets, while 54.1% (*n* = 253) responded with “Yes” on the item about diets containing high-quality protein, and 55.1% (*n* = 258) were aware about the foods they should avoid. Another 59.2% (*n* = 277) were aware about the appropriate daily salt usage for their condition. The majority of the respondents, 49.8% (*n* = 233), were unaware about laboratory examinations, which they should regularly check, while 59.6% (*n* = 279) responded with “No” or “Don’t know” on the item about understanding of test reports; i.e., the other participants claimed to have the understanding to interpret their laboratory tests.

### 3.4. Awareness Regarding the Management of the Disease Condition

The following table (Table 5) summarizes respondents’ awareness of the management of CKD: 57.9% (*n* = 271) were aware of the importance of exercise for fitness, and more than half of participants (51.1%, *n* = 239) were aware of the evaluation of curative effects of disease, which means they were aware of signs and symptoms that meant the improvement of their health; 62.8% (*n* = 294) of patients were aware of how to contact a physician in case of query regarding the condition of the disease.

### 3.5. Association between Demographic Characteristics and Awareness Regarding CKD (p-Value by Chi-Square Analysis)

For inferential analysis, the chi-square test was used to ascertain the association between the responses to the CKD-specific questions (items 1–18) and socio-demographic characteristics and clinical/patient conditions (Table 6). Most items have no strong association in case of stage, duration of CKD, and patient type. All items were highly associated with education level except for the statement corresponding to the awareness regarding salt usage. Higher level of education showed good awareness for most responses. Age group and male gender awareness was also associated in most of the cases, with better awareness shown in more advanced age groups.

## 4. Discussion

Disease knowledge and awareness are critical educational components that can aid individuals in improving their ability to control their own health. CKD self-care is complicated and requires patients to keep track of their disease (e.g., monitoring their blood pressure and weight as well as their body temperature and cholesterol level), fluid intake management, and diet; engage in physical activity; adhere to medication regimens; comprehend new information; and communicate with health-care practitioners and other patients. This survey was the first of its nature in the city of Quetta, and it was conducted among patients impacted by the fields of nephrology and urology. The findings of this study revealed that, in general, people’s level of awareness, in the majority of cases, was extremely low, which is extremely concerning not only for public health but also for the government authorities due to the disease burden on the country’s already suffering economy.

A higher prevalence of chronic kidney disease is also associated with social deprivation [33]. There were correlations observed between risk factors for chronic renal disease, such as smoking, drinking alcohol, increasing belly circumference (obesity), and elderly awareness of the condition [34]. Diabetic nephropathy has emerged as the leading cause of kidney disease in India, according to a new study [35]. Chronic diseases, particularly chronic kidney disease, have received less attention owing partly to the global health community’s focus on infectious diseases and a lack of awareness. There is an urgent need for funding in and to poor nations to undertake more comprehensive, cost-effective, and preventative chronic illness interventions [36].

Our study highlighted that the awareness of patients regarding CKD is very low in some areas, and the majority of participants were unaware about the progression of the disease and the acts that may aggravate their kidney functions further. In addition, they were not aware of the symptoms that may appear due to worsening of the disease and when to seek help from HCPs. Our study is in agreement with previous studies conducted in the United States [37,38,39], Australia [29], Brazil [40], Hong Kong [41], Ethiopia [42,43], Nigeria [44,45], Tanzania [46], Singapore [47], Iran [48], and India [49]. The summary of the main findings of the above few studies are listed in Table 7. The majority of graduate respondents in our study reported a good understanding about controlling their blood pressure when elevated, while respondents who had no formal education or had primary education were not aware of how to do so. Education showed a significant association with knowledge on controlling blood pressure. Further studies on this area of awareness and control are desperately needed to explore further the understanding of patients affected by this disease in Pakistan.

Men were more abundant among responders, and because the study was time-limited, one probable explanation is that male patients were more prevalent during that period. Yet, in 2016, there were 752.7 million people worldwide with impaired kidney function, including 417.0 million females and 335.7 million males (female-to-male ratio: 1.24) [50]. Previous study in Pakistan reported that 56.5% of (*n* = 255 CKD) patients were males, and 43.5% were females, indicating a significant proportion of male respondents [51]. The global burden of illness study reveals significant gender disparities in the prevalence of CKD across 195 nations. However, the nature of these discrepancies is complex and must be evaluated cautiously, considering all conceivable local and general conditions.

The general age is of our sample was relatively young (around 30 years); possible reasons for this could be that this patient group can reach the healthcare facilities more easily, and they are more health conscious. Individuals with a higher level of education are more likely to attend healthcare facilities, whereas access to healthcare, lifestyle choices, and coexisting conditions all likely contribute to the association between low educational attainment and the frequency of chronic kidney disease in young adults [52]. End-stage kidney disease (ESKD) is frequently an illness of youth in Pakistan. Although Pakistan does not have a comprehensive national registry for ESKD, studies indicate that the median age of patients approaching ESKD in Pakistan is 44 years [53] compared to 63 years in the United States [54], most likely due to a lack of routine medical care [55]. Mortality rates are particularly high due to a shortage of inexpensive dialysis [56]. Only 10% of patients with ESKD are able obtain dialysis, and the majority of those who do die within three months of starting dialysis [53]. A previous study indicated 46% of respondents favored a treatment strategy that prioritized comfort and symptom management over life extension [57].

The respondents had good understanding on the fact that living a full life with one healthy kidney was possible, and this knowledge level was higher in the patients in the later stages of the disease. Previous studies suggest that differences in views regarding the features of transplantation between rural and urban patients have been observed, which may contribute to geographic inequities in transplant recipients, subsequent therapeutic success, and QoL [58]. These findings could be used to inform future-tailored, culturally appropriate transplant education treatments for CKD patients. Self-reported awareness related to the names and primary roles (i.e., indications) of medications was reportedly very low, which is similar to the findings of a previous study done in Hong Kong, where the respondents had an inadequate understanding about CKD in addition to having noticeable gaps in knowledge regarding the pharmacological treatment of this disease [41]. The majority of the patients were aware of herbal medicines, and when inquired, most of the respondents believed that a few of them, such as garlic (leaf), papaya (leaf, fruit), turmeric (rhizome), black mustard (leaf), and coriander (leaf), had positive or better effects on the kidney. The relationship between these findings and education level was significant. This could be related to the high prevalence of complementary and alternative medicine (CAM) usage in the area, and these findings were consistent with earlier studies [41,59,60].

Reduced prevalence of CKD and albuminuria is linked to a balanced dietary pattern rich in vegetables, fruit, legumes, nuts, whole grains, fish, and low-fat dairy as well as the decreased intake of red and processed meats, sodium, and sugar-sweetened beverages [61]. In the present healthcare system, patients are usually told about the dietary risks after every checkup, and the information about diet risks are disseminated through all HCPs multiple times. This could be a potential reason for our study results, as it showed good awareness of patients regarding dietary risks, which included unhealthy dietary patters, food containing high-quality protein, and a list of foods which should be avoided. Awareness of patients regarding laboratory examinations and understanding of these reports were shown to be poor; the majority of the patients were not aware about which laboratory tests they should check regularly. A previous study regarding the awareness of physicians about laboratory investigations in CKD was conducted in 2005 [62], which concluded poor awareness in these HCPs as well. The reason of low patient awareness may be due to the low literacy rate (both general and numerical) in our society [63], as our study showed a significant relationship between awareness of laboratory investigations with education level.

According to the study, more than half of the respondents knew how much salt they should consume on daily basis; previous studies have shown that people are becoming more aware of salt varieties and its daily recommended usage [64,65]. Salt usage awareness was high in older age groups due to broader dissemination of knowledge through healthcare professionals, personal experience, the media, and national food companies in addition to the fact that similar recommendations are usually made for hypertension as well. Improving knowledge on the curative affect (improvement in disease condition) and increasing the confidence of the affected patients to ask their caregivers whenever and whatever problem arises is the goal of many civil and professional organizations working on CKD [3,66]. While more research is needed in this area, it is possible that the reason for good patient knowledge of therapeutic effects and fitness activity is due to caregivers’ guidance, improved counseling, or the patient’s health consciousness.

When the findings of the study are applied to the wider population, they may even worsen the situation. Because of the scarcity of healthcare facilities in rural areas, many patients in need of specialized care in nephrology and urology do not have access to such care. Those patients can also be deemed unaware of their situation. Although the exact number of such cases is unknown, it is likely to be higher in numbers, making the conclusions of the study even more concerning and necessitating prompt action.

### Limitations of the Study

The limitations of the present study should be acknowledged, including the cross-sectional nature and convenience sampling design for data collection. One of the limitations of the study is the non-availability of data and studies pertaining to the prevalence of CKD in the community. Our study aimed to measure the self-reported understanding of affected patients; however, this may not reflect entirely the real knowledge of the participants. Moreover, the limited period for data collection was also an obstacle in the study. The emergence of the novel coronavirus pandemic (SARS-CoV-2) and COVID-19 standard operating procedures (SOPs) in healthcare institutions have limited the access to patients and thus larger numbers of responses. The findings of these studies are of a single center (although a clinically significant setting) of the Nephrology and Urology Institute in Quetta. For a more extensive and better understanding, the same study and data collection should be performed in other relevant institutes of Pakistan as well.

## 5. Conclusions

Based on the results of our study, the level of awareness regarding the symptomatology, medications, and appropriate lifestyle for CKD among nephrology and urology patients of Quetta was considerably low. In addition, the majority of affected patients in the region has limited understanding about the laboratory investigations for CKD, the context, and the interpretation of these results for patients. The study’s main findings are quite disturbing and require immediate attention from public health officials in order to reduce the burden of disease on the government and people.

## 6. Suggestions and Recommendations

Disseminating information about kidney diseases in the public (as a part of novel or existing health promotion campaigns) may improve the awareness among patients and community. For instance, seminars and educations programs can be arranged to help the public to understand kidney problems in settings with a high turnover of people. Medication agencies and the media may also play important roles to inform the public about kidney issues. Improving public knowledge and awareness will lead to proper management and early detection of the disease, leading to better patient health outcome; morbidity and mortality rates will be reduced, and national disease burden will be altered in an advantageous direction (by relieving the healthcare infrastructure mediated by the government and private organizations) if the community is well-informed about CKD and its onset.

## Figures and Tables

**Table 1:** Reliability analysis of the instrument.

	Scale Mean if Item Deleted	Scale Variance if Item Deleted	Corrected Item-Total Correlation	Squared Multiple Correlation	Cronbach’s Alpha if Item Deleted
Item 1	10.86	37.495	0.434	0.540	0.841
Item 2	10.66	36.069	0.476	0.591	0.840
Item 3	10.79	37.974	0.415	0.469	0.842
Item 4	10.87	37.777	0.403	0.320	0.843
Item 5	10.98	38.050	0.345	0.312	0.846
Item 6	10.84	37.339	0.540	0.470	0.837
Item 7	10.83	37.156	0.511	0.467	0.838
Item 8	10.51	36.754	0.482	0.348	0.839
Item 9	10.70	38.736	0.277	0.239	0.849
Item 10	10.85	36.910	0.551	0.463	0.836
Item 11	10.81	37.029	0.472	0.404	0.839
Item 12	10.85	36.519	0.544	0.422	0.836
Item 13	10.92	38.273	0.333	0.274	0.846
Item 14	10.90	38.135	0.362	0.343	0.845
Item 15	10.65	38.039	0.390	0.308	0.843
Item 16	10.71	37.829	0.521	0.425	0.838
Item 17	10.86	36.489	0.518	0.423	0.837
Item 18	10.86	36.388	0.523	0.452	0.837

**Table 2:** Demographic characteristics of the participants.

	Responses	Frequency (*n*, %)
Gender	Male	347 (74.1%)
	Female	121 (25.9%)
Age Groups	Below 15 years	3 (0.6%)
	15–25 years	190 (40.6%)
	26–35 years	174 (37.2%)
	36–45 years	56 (12.0%)
	46–55 years	26 (5.6%)
	Above 55 years	19 (4.1%)
Education Level	No education	53 (11.3%)
	Primary school	50 (10.7%)
	Secondary school	80 (17.1%)
	Intermediate	91 (19.4%)
	Graduate	139 (29.7%)
	Postgraduate	22 (4.7%)
	Religious education	33 (7.1%)
Patient Type	Inpatient	211 (45.1%)
	Outpatient	246 (52.6%)
	Dialysis patient	11 (2.4%)

**Table 3:** Clinical profile and lifestyle factors of the participants.

	Responses	Frequency (*n*, %)
Duration of CKD	1–3 years	346 (73.9%)
	3–5 years	78 (16.7%)
	>5 years	44 (9.4%)
Comorbidities	Diabetes mellitus	54 (8.8%)
	Hypertension	129 (21.0%)
	Other cardiovascular diseases	64 (10.4%)
	Chronic emotional Stress	163 (26.6%)
	No other comorbidities	203 (33.1%)
Lifestyle Factors	Smoking	147 (31.4%)
	Non-smoker	321 (68.6%)
	Inadequate exercise (<30 min/day)	194 (41.5%)
	Exercise (≥30 min/day)	274 (58.5%)
CKD Stage	Stage 1 (GFR 90 mL/min/1.73 m^2^ or higher)	354 (75.6%)
	Stage 2 (GFR 60–89 mL/min/1.73 m^2^)	74 (15.8%)
	Stage 3 (GFR 30–59 mL/min/1.73 m^2^)	25 (5.3%)
	Stage 4 (GFR 15–29 mL/min/1.73 m^2^)	13 (2.8%)
	Stage 5 (GFR less than 15 mL/min/1.73 m^2^)	2 (0.4%)

**Table 4:** Awareness of the participants regarding the CKD disease condition and associated factors.

Item	Response	
	Yes (*n*, %)	No and Don’t Know (*n*, %)
Do you know how to control your blood pressure (BP)?	284 (60.7%)	184 (39.4%)
Did you know that a person may lead a normal life with one healthy kidney?	306 (65.4%)	162 (34.6%)
Do you know what symptoms will develop when your condition gets worse?	233 (49.8%)	235 (50.3%)
Do you know what aggravates your kidney function?	205 (43.8%)	263 (56.2%)
Do you know the long-term prognosis of your disease?	205 (43.8%)	263 (56.2%)
Do you know the brand names and usages of your medicines?	227 (48.5%)	241 (51.5%)
Do you know the primary role of your medicines?	208 (44.4%)	260 (55.5%)
Do you know which medicines may impair your kidney function?	176 (37.6%)	292 (62.4%)
Do you know which herbal supplements can be effective in treating chronic kidney disease?	199 (42.5%)	269 (57.5%)
Do you know what unhealthy diets are?	243 (51.9%)	225 (48.1%)
Do you know which food contains high-quality protein?	253 (54.1%)	215 (46.0%)
Do you know which food should be avoided in your condition?	258 (55.1%)	210 (44.9%)
Do you know how much salt you should be using daily?	277 (59.2%)	191 (40.8%)
Do you know what laboratory examinations you should regularly check to track your disease condition?	156 (33.3%)	312 (66.7%)
Do you know the meaning of your test reports and their interpretation?	143 (30.6%)	325 (69.4%)

**Table 5:** Awareness of the participants regarding management of the CKD disease condition.

Item	Response	
	Yes (*n*, %)	No and Don’t Know (*n*, %)
Do you know what exercise fits you?	271 (57.9%)	197 (42.1%)
Do you know how to evaluate your curative effect?	239 (51.1%)	229 (48.9%)
Do you know how/where to contact healthcare professionals when you have a question regarding your illness?	294 (62.8%)	174 (37.2%)

**Table 6:** *p*-Value by chi-square analysis.

Items	Age Group	Gender	Education Level	Duration of CKD	Patient Type	Stage of CKD
Item 1	***0.002***	***0.001***	***0.001***	0.057	**0.037**	0.658
Item 2	**0.021**	***0.001***	***0.006***	0.110	***0.001***	0.294
Item 3	0.432	0.266	**0.013**	0.203	0.438	0.522
Item 4	**0.017**	***0.001***	***0.001***	0.130	0.999	***0.004***
Item 5	0.065	***0.001***	***0.001***	0.274	0.398	**0.041**
Item 6	0.099	0.141	***0.001***	0.526	**0.036**	0.267
Item 7	0.103	**0.015**	***0.001***	0.495	**0.041**	0.489
Item 8	***0.001***	0.072	***0.001***	0.503	0.309	**0.017**
Item 9	***0.001***	0.614	***0.006***	***0.003***	0.273	0.159
Item 10	**0.01**	0.111	***0.002***	***0.008***	0.215	0.386
Item 11	***0.001***	**0.03**	***0.001***	***0.001***	0.804	0.524
Item 12	0.063	0.792	***0.001***	0.316	**0.04**	0.462
Item 13	***0.002***	0.908	0.283	***0.003***	0.070	0.343
Item 14	***0.001***	***0.002***	***0.001***	***0.001***	0.332	0.410
Item 15	0.133	**0.015**	**0.01**	0.779	0.243	0.555
Item 16	0.099	**0.017**	***0.001***	0.342	0.528	0.980
Item 17	0.281	***0.001***	***0.001***	0.104	0.624	0.879
Item 18	***<0.001***	***<0.001***	***<0.001***	0.462	0.350	***0.002***

Values in **boldface** represent *p*-values < 0.05, while values in ***boldface and italics*** represent *p*-values < 0.01.

**Table 7:** Previous findings about CKD knowledge and awareness.

First Author, Year of Publication	Country	Main Findings
W. M. McClellan et al., 2009	Georgia (USA)	In the U.S. community of older persons with or without coronary heart disease, there was a significant prevalence of CKD and a low prevalence of renal disease awareness. These findings back up previous suggestions that individuals with cardiovascular illness should be evaluated for CKD and given information about it.
Gheewala et al., 2018	Australia	The general public in Australia had a difficult time understanding CKD. People in Australia should be educated more about this illness, which could lead to better detection and management of the disease.
Bezerra da Silva Junior et al., 2020	Brazil	CKD awareness in country appears to be on the rise, with a large number of people asked claiming to know how to prevent it, but this is still a far cry from an optimal situation. Goals for CKD prevention and treatment seem to be achievable with the use of healthcare technology.
K. Chow et al., 2014	Hong Kong	CKD is largely unknown in Hong Kong, and there are significant gaps in understanding concerning the role of hypertension in the development of renal illness.
Okoro et al., 2020	Nigeria	According to the findings, the majority of people with CKD had limited awareness about the disease. A person’s tertiary educational degree was the only significant independent predictor of their improved CKD knowledge.
W. L. Chow et al., 2012	Singapore	Older patients with lower educational and socioeconomic status should be targeted for CKD education.
Roomizadeh et al., 2014	Iran	The community has little knowledge of CKD and its risk factors. Future public health education campaigns should emphasize the significance of regular renal care counseling and educating the Iranian people about the asymptomatic character of CKD in its early stages.
Hussain et al., 2019	India	According to the findings of this study, Indian patients have a poor understanding of CKD. In order to deal with this developing co-morbid illness, the government should implement CKD awareness initiatives.

## Data Availability

All data generated during the study are presented in this paper.

## Supplementary Materials

The following supporting information can be downloaded at: https://www.mdpi.com/article/10.3390/ijerph19095015/s1. Supplementary Material S1: Final version of the data collection tool.

## References

[1] Levey A.S., Coresh J., Balk E., Kausz A.T., Levin A., Steffes M.W., Hogg R.J., Perrone R.D., Lau J., Eknoyan G. (2003). National Kidney Foundation Practice Guidelines for Chronic Kidney Disease: Evaluation, Classification, and Stratification. Ann. Intern. Med..

[2] Walker R. (2011). Clinical Pharmacy and Therapeutics E-Book.

[3] Narva A.S., Briggs M. (2009). The National Kidney Disease Education Program: Improving understanding, detection, and management of CKD. Am. J. Kidney Dis..

[4] Bikbov B., Purcell C.A., Levey A.S., Smith M., Abdoli A., Abebe M., Adebayo O.M., Afarideh M., Agarwal S.K., Agudelo-Botero M. (2020). Global, regional, and national burden of chronic kidney disease, 1990–2017: A systematic analysis for the Global Burden of Disease Study 2017. Lancet.

[5] Imtiaz S., Salman B., Qureshi R., Drohlia M., Ahmad A. (2018). A review of the epidemiology of chronic kidney disease in Pakistan: A global and regional perspective. Saudi J. Kidney Dis. Transplant..

[6] Jacobson S. (2013). Chronic kidney disease—A public health problem?. Lakartidningen.

[7] Brasil (2014). Diretrizes clínicas para o cuidado ao paciente com doença renal crônica-DRC no Sistema Único de Saúde. Secr. Aten. Saúde.

[8] Cruz C.F., Cunha G., Souza S.J.S.H. (2014). Cost of treatment of patients with chronic renal failure end stage in São Paulo in the period from 2008 to 2012. Sci. Health.

[9] Lv J.-C., Zhan L.-X. (2019). Prevalence and Disease Burden of Chronic Kidney Disease. Adv. Exp. Med. Biol..

[10] Zelle D.M., Klaassen G., van Adrichem E., Bakker S.J., Corpeleijn E., Navis G. (2017). Physical inactivity: A risk factor and target for intervention in renal care. Nat. Rev. Nephrol..

[11] Jain N., Reilly R.F. (2014). Effects of dietary interventions on incidence and progression of CKD. Nat. Rev. Nephrol..

[12] Tillyard G., DeGennaro V. (2019). New methodologies for global health research: Improving the knowledge, attitude, and practice survey model through participatory research in Haiti. Qual. Health Res..

[13] Jordan J.E., Buchbinder R., Osborne R. (2010). Conceptualising health literacy from the patient perspective. Patient Educ. Couns..

[14] Mulango I.D., Atashili J., Gaynes B.N., Njim T. (2018). Knowledge, attitudes and practices regarding depression among primary health care providers in Fako division, Cameroon. BMC Psychiatry.

[15] Chen J., Zhao H., Xia Z., Zhang Y., Lv X., Zhou X., Dong X., Li J., Jiang H., Huang Y. (2018). Knowledge, attitude, and practice toward the daily management of PICC in critically ill cancer patients discharged from intensive care units. Cancer Manag. Res..

[16] Peng S., He J., Huang J., Tan J., Liu M., Liu X., Wu Y. (2019). A chronic kidney disease patient awareness questionnaire: Development and validation. PLoS ONE.

[17] Kayyali R., Gebara S.N., Hesso I., Funnell G., Naik M., Mason T., Uddin M.A., Al-Yaseri N., Khayyam U., Al-Haddad T. (2018). Shared decision making and experiences of patients with long-term conditions: Has anything changed?. BMC Health Serv. Res..

[18] Navaneethan S.D., Aloudat S., Singh S. (2008). A systematic review of patient and health system characteristics associated with late referral in chronic kidney disease. BMC Nephrol..

[19] Saleem F., Hassali M.A., Shafie A.A., Awad A.G., Bashir S. (2011). Association between knowledge and drug adherence in patients with hypertension in Quetta, Pakistan. Trop. J. Pharm. Res..

[20] Haq N., Tahir M., Iqbal Q., Naseem Q. (2015). Exploration of osteoporosis knowledge and perception among young women in Quetta, Pakistan. J. Osteopor. Phys. Act..

[21] Zafar T., Brahmbhatt H., Imam G., Hassan S.U., Strathdee S.A. (2003). HIV Knowledge and Risk Behaviors Among Pakistani and Afghani Drug Users in Quetta, Pakistan. JAIDS J. Acquir. Immune Defic. Syndr..

[22] Haq N., Khan Z., Riaz S., Nasim A., Shahwani R., Tahir M. (2017). Prevalence and knowledge of polycystic ovary syndrome (PCOS) among female science students of different public Universities of Quetta, Pakistan. Imp. J. Interdiscip. Res..

[23] Haq N.U., Hassali M.A., Shafie A.A., Saleem F., Farooqui M., Haseeb A., Aljadhey H. (2013). A cross-sectional assessment of knowledge, attitude and practice among Hepatitis-B patients in Quetta, Pakistan. BMC Public Health.

[24] Khan A.S., Khan S.D., Kakar D.M. (2013). Land subsidence and declining water resources in Quetta Valley, Pakistan. Environ. Earth Sci..

[25] National Institute of Population Studies—NIPS/Pakistan, The DHS Program, ICF (2019). Pakistan Demographic and Health Survey 2017–18.

[26] Sabzwari S.R. (2017). Health literacy in Pakistan: Exploring new ways of addressing an old challenge. J. Pak. Med. Assoc..

[27] BINUQ Balochistan Institute of Nephro-Urology Quetta. https://www.binuq.gob.pk/.

[28] Raosoft, Inc. (2004). RaoSoft Sample Size Calculator. http://www.raosoft.com/samplesize.html.

[29] Gheewala P.A., Peterson G.M., Zaidi S.T.R., Jose M.D., Castelino R.L. (2018). Public knowledge of chronic kidney disease evaluated using a validated questionnaire: A cross-sectional study. BMC Public Health.

[30] Son J. (2018). Back translation as a documentation tool. Transl. Interpret..

[31] Maneesriwongul W., Dixon J.K. (2004). Instrument translation process: A methods review. J. Adv. Nurs..

[32] Amir S. (2019). English Language Teaching and Cultural Implications: An Analysis of Higher Secondary School Subject Specialists’ Perceptions and Practices in Pakistan. Prax. Int. J. Soc. Sci. Lit..

[33] McKinley J.M., Mueller U., Atkinson P.M., Ofterdinger U., Cox S.F., Doherty R., Fogarty D., Egozcue J.J., Pawlowsky-Glahn V. (2020). Chronic kidney disease of unknown origin is associated with environmental urbanisation in Belfast, UK. Environ. Geochem. Health.

[34] Delgado M.F., Lisboa I.N.D., Fernandes M.I.D.C.D., Carino A.C.C., Fernandes R.M., Lira A.L.B.D.C. (2017). Risk factors and knowledge of the elderly people about chronic kidney disease. Rev. Rede Enferm. Nord..

[35] Rajapurkar M.M., John G.T., Kirpalani A.L., Abraham G., Agarwal S.K., Almeida A.F., Gang S., Gupta A., Modi G., Pahari D. (2012). What do we know about chronic kidney disease in India: First report of the Indian CKD registry. BMC Nephrol..

[36] Nugent R.A., Fathima S.F., Feigl A.B., Chyung D. (2011). The Burden of Chronic Kidney Disease on Developing Nations: A 21st Century Challenge in Global Health. Nephron Clin. Pract..

[37] McClellan W.M., Newsome B.B., McClure L.A., Cushman M., Howard G., Audhya P., Abramson J.L., Warnock D.G. (2009). Chronic Kidney Disease Is Often Unrecognized among Patients with Coronary Heart Disease: The REGARDS Cohort Study. Am. J. Nephrol..

[38] Tuot D.S., Plantinga L.C., Hsu C.-Y., Jordan R., Burrows N.R., Hedgeman E., Yee J., Saran R., Powe N.R., for the Centers for Disease Control Chronic Kidney Disease Surveillance Team (2011). Chronic Kidney Disease Awareness Among Individuals with Clinical Markers of Kidney Dysfunction. Clin. J. Am. Soc. Nephrol..

[39] Kazley A.S., Johnson E., Simpson K., Chavin K., Baliga P. (2014). African American patient knowledge of kidney disease: A qualitative study of those with advanced chronic kidney disease. Chronic Illn..

[40] Junior G.B.D.S., Oliveira M.J.C., Pedrosa A.S.S., Pereira G.A., Vilela I.H.P., Veloso F.C.S., De Oliveira L.L., Oliveira F.M., Jordão D.A., Costa A.B.A.L. (2020). Knowledge about chronic kidney disease and the ways of prevention: A population-based study in northeast Brazil. Nephrol. Dial. Transplant..

[41] Chow K., Szeto C., Kwan B.C., Leung C., Li P.K. (2014). Public lacks knowledge on chronic kidney disease: Telephone survey. Hong Kong Med. J..

[42] Asmelash D., Chane E., Desalegn G., Assefa S., Aynalem G.L., Fasil A. (2020). Knowledge and Practices towards Prevention and Early Detection of Chronic Kidney Disease and Associated Factors among Hypertensive Patients in Gondar Town, North West Ethiopia. Int. J. Hypertens..

[43] Yabeyu A.B., Haile K., Belay Y., Tegegn H.J.A.P. (2021). Public Knowledge of Chronic Kidney Disease in a Resource-Limited Setting: A Cross-Sectional Study. Authorea Prepr..

[44] Okoro R.N., Ummate I., Ohieku J.D., Ibn Yakubu S., Adibe M.O., Okonta M.J. (2020). Kidney Disease Knowledge and Its Determinants Among Patients with Chronic Kidney Disease. J. Patient Exp..

[45] Akpan E.E., Ekrikpo U.E. (2015). Chronic kidney failure: Knowledge of kidney disease, perception of causes and symptomatology in Uyo, Nigeria. Open J. Nephrol..

[46] Stanifer J.W., Turner E.L., Egger J.R., Thielman N., Karia F., Maro V., Kilonzo K., Patel U.D., Yeates K. (2016). Knowledge, Attitudes, and Practices Associated with Chronic Kidney Disease in Northern Tanzania: A Community-Based Study. PLoS ONE.

[47] Chow W.L., Joshi V.D., Tin A.S., Van der Erf S., Lim J.F., Swah T.S., Teo S.S., Goh P.S., Tan G.C., Lim C. (2012). Limited knowledge of chronic kidney disease among primary care patients–a cross-sectional survey. BMC Nephrol..

[48] Roomizadeh P., Taheri D., Abedini A., Mortazavi M., Larry M., Mehdikhani B., Mousavi S.-M., Hosseini F.-A., Parnia A., Nakhjavani M. (2014). Limited knowledge of chronic kidney disease and its main risk factors among Iranian community: An appeal for promoting national public health education programs. Int. J. Health Policy Manag..

[49] Hussain S., Habib A., Najmi A.K. (2019). Limited knowledge of chronic kidney disease among type 2 diabetes mellitus patients in India. Int. J. Environ. Res. Public Health.

[50] Bikbov B., Perico N., Remuzzi G., on behalf of the GBD Genitourinary Diseases Expert Group (2018). Disparities in Chronic Kidney Disease Prevalence among Males and Females in 195 Countries: Analysis of the Global Burden of Disease 2016 Study. Nephron Exp. Nephrol..

[51] Chand S., Khan G.R. (2021). Evaluation of Health Related Quality of Life in Patients with Chronic Kidney Disease. Asian J. Allied Health Sci..

[52] Tripathy S., Cai X., Adhikari A., Kershaw K., Peralta C.A., Kramer H., Jacobs D.R., Gutierrez O.M., Carnethon M.R., Isakova T. (2020). Association of educational attainment with incidence of CKD in young adults. Kidney Int. Rep..

[53] Sakhuja V., Sud K. (2003). End-stage renal disease in India and Pakistan: Burden of disease and management issues. Kidney Int..

[54] Foley R.N., Collins A.J. (2007). End-stage renal disease in the United States: An update from the United States Renal Data System. J. Am. Soc. Nephrol..

[55] Sakhuja V., Kohli H. (2006). End-stage renal disease in India and Pakistan: Incidence, causes, and management. Ethn. Dis..

[56] Jha V. (2009). Current status of end-stage renal disease care in South Asia. Ethn. Dis..

[57] Saeed F., Sardar M., Rasheed K., Naseer R., Epstein R.M., Davison S.N., Mujtaba M., Fiscella K.A. (2020). Dialysis Decision Making and Preferences for End-of-Life Care: Perspectives of Pakistani Patients Receiving Maintenance Dialysis. J. Pain Symptom Manag..

[58] Ghahramani N., Wang C., Sanati-Mehrizy A., Tandon A. (2014). Perception About Transplant of Rural and Urban Patients with Chronic Kidney Disease; A Qualitative Study. Nephro-Urol. Mon..

[59] Rani N.V., Rao A.S.M.A., Phaneendra D., Pavani C.D., Soundararajan P., Thennarasu P., Kannan G. (2016). Usage of complementary and alternative medicine among patients with chronic kidney disease on maintenance hemodialysis. J. Pharm. Bioallied Sci..

[60] Jha V. (2010). Herbal medicines and chronic kidney disease. Nephrology.

[61] Bach K.E., Kelly J.T., Palmer S.C., Khalesi S., Strippoli G.F., Campbell K.L. (2019). Healthy dietary patterns and incidence of CKD: A meta-analysis of cohort studies. Clin. J. Am. Soc. Nephrol..

[62] Stevens L.A., Fares G., Fleming J., Martin D., Murthy K., Qiu J., Stark P.C., Uhlig K., Van Lente F., Levey A.S. (2005). Low Rates of Testing and Diagnostic Codes Usage in a Commercial Clinical Laboratory: Evidence for Lack of Physician Awareness of Chronic Kidney Disease. J. Am. Soc. Nephrol..

[63] Khan F.Z., Kamran M., Saeed A.A.R., Faizi W.U.N. (2021). Barriers responsible to females’ low literacy at the primary level in balochistan province of pakistan: Social and economic barriers. Humanit. Soc. Sci. Rev..

[64] Lowe N., Westaway E., Munir A., Tahir S., Dykes F., Lhussier M., McKeown M., Zimmerman M., Andersson M., Stinca S. (2015). Increasing Awareness and Use of Iodised Salt in a Marginalised Community Setting in North-West Pakistan. Nutrients.

[65] Khan G.N., Hussain I., Soofi S.B., Rizvi A., Bhutta Z.A. (2012). Study on the Household Use of Iodised Salt in Sindh and Punjab Provinces, Pakistan: Implications for Policy Makers. J. Pharm. Nutr. Sci..

[66] Narva A.S., Norton J.M., Boulware L.E. (2015). Educating Patients about CKD: The Path to Self-Management and Patient-Centered Care. Clin. J. Am. Soc. Nephrol..

