# Supplementary material for: Awareness of Chronic Kidney Disease, Medication, and Laboratory Investigation among Nephrology and Urology Patients of Quetta, Pakistan
Source: Int J Environ Res Public Health. 2022 Apr 20;19(9):5015. doi: 10.3390/ijerph19095015 (PMC9103555; doi:10.3390/ijerph19095015)
Supplement: Supplementary file 1 [file ijerph-19-05015-s001.zip › ijerph-1626962-supplementary.pdf]

Data collection form: ____

Awareness of Chronic Kidney Disease; its medication and laboratory investigation among Nephrology & Urology patients of Quetta.

QUESTIONNAIRE سوالنامہ

Please tick (✓) your answer to the following questions.

براہ کرم درج ذیل سوالات کے اپنے جواب پر(✓) نشان لگائیں

1. Age عمر: ____ 2. Gender جنس : ☐Male مرد ☐Female عورت

3. Education تعلیم : ☐Primary School پرائمری اسکول ☐Secondary School ثانوی اسکول

☐Intermediate انٹرمیڈیٹ ☐Graduate گریجویٹ

☐Post Graduate پوسٹ گریجویٹ ☐Religious Education دینی تعلیم

☐No Education کوئ تعلیم نہیں

3. Do you have any of the following disease? / کیا آپ کو مندرجہ ذیل میں سے کوئی بیماری ہے؟

☐Diabetes mellitus ذیابیطس ☐Hypertension ہائی بلڈ پریشر

☐Cardiovascular disease دل کی بیماری ☐Stress ذہنی دباؤ

☐No Disease کوئ بیماری نہیں

4. Duration of Disease بیماری کی مدت:

☐0-3 Years ☐3-5 Years ☐More than Five years

5. Life Style: طرز زندگی

- Do you Smoke? کیا آپ سگریٹ نوشی کرتے ہیں؟

  ☐Yes ہاں ☐No نہیں

- Do you exercise 30 mins per day? کیا آپ روزانہ تیس منٹ ورزش کرتے ہیں؟

  ☐Yes ہاں ☐No نہیں

Data collection form: ____

6. CKD Stages: ☐Stage 1 (GFR 90 or Greater) ☐Stage 2 (GFR Between 60 and 89)

☐Stage 3 (GFR Between 30 and 59) ☐Stage 4 (GFR Between 15 and 29)

☐Stage 5 (GFR Less than 15)

**Please tick (✓) your answer to the following questions.**	Yes<br>ہاں	No<br>نہیں	Don't Know<br>پتہ نہیں
1. Do you know how to control your blood pressure (BP)?<br>کیا آپ جانتے ہیں کہ آپ اپنے بلڈ پریشر (بی۔پی) کو کس طرح کنٹرول کر سکتے ہیں؟	☐	☐	☐
2. Do you know that person may lead a normal life with one healthy kidney?<br>کیا آپ جانتے ہیں کہ انسان صرف ایک صحت مند گردے کے ساتھ تندرست زندگی گزار سکتا ہے؟	☐	☐	☐
3. Do you know what symptoms will develop when you get worse?<br>کیا آپ جانتے ہیں کہ آپکی بیماری کے بگڑنے کی صورت میں کون سی علامات پیدا ہوں گی؟	☐	☐	☐
4. Do you know what aggravates your kidney function?<br>کیا آپ جانتے ہیں کہ آپ کے گردے کی خرابی کن عوامل سے بڑھ جاتی ہے؟	☐	☐	☐
5. Do you know the long-term prognosis of your disease?<br>کیا آپ کو اپنے مرض کی طویل المیعاد پیچیدگیوں کے بارے میں معلوم ہے؟	☐	☐	☐
6. Do you know the brand names and usage of your medicines?<br>کیا آپ اپنی دوائیوں کے نام اور استعمال سے واقف ہیں؟	☐	☐	☐
7. Do you know the primary role of your medicines?<br>کیا آپکو معلوم ہے کہ آپکو دی جانے والی ادویات کیوں دی گئی ہیں؟	☐	☐	☐
8. Do you know which medicine may impair the kidney function?<br>کیا آپ جانتے ہیں کہ آپکو دی جانے والی دویات گردوں پہ منفی اثر مرتب کر سکتی ہیں؟	☐	☐	☐
9. Do you know Herbal supplements can be effective in treating chronic kidney disease?<br>کیا آپ جانتے ہیں کہ یونانی ادویات گردوں کے دائمی مرض کیلیے مفید ہو سکتی ہیں؟	☐	☐	☐

Data collection form: ____

Please tick (✓) your answer to the following questions.	Yes<br>ہاں	No<br>نہیں	Don't Know<br>پتہ نہیں
10. Do you know what unhealthy diets are?<br>کیا آپ کو ان غزاؤں کے متعلق علم ہے جو گردوں پر اثرانداز ہوں؟	☐	☐	☐
11. Do you know which food contains high-quality protein?<br>کیا آپ کو ان غزاؤں کے متعلق علم ہے جن میں پروٹین کی مقدار زیادہ ہو؟	☐	☐	☐
12. Do you know food, which should be avoided, in your condition?<br>کیا آپ کو ان غزاؤں کے متعلق علم ہے جن سے آپکو اپنی بیماری کی وجہ سے اجتناب کرنا چاہیئے؟	☐	☐	☐
13. Do you know how much salt you should be using daily?<br>کیا آپ جانتے ہیں کہ اس بیماری میں نمک کا کم استعمال کرنا چاہیئے؟	☐	☐	☐
14. Do you know what laboratory examinations you should regularly check?<br>کیا آپ جانتے ہیں کہ آپ کو کون سے لیبارٹری ٹیسٹ باقاعدگی سے کرانے چاہئیں؟	☐	☐	☐
15. Do you know the meaning of your test reports?<br>کیا آپ اپنے کیے جانے والے لیبارٹری ٹیسٹ کے نتائج کو سمجھ سکتے ؟	☐	☐	☐
16. Do you know what exercise fits you?<br>کیا آپ جانتے ہیں کہ کون سی جسمانی ورزش آپ کے لیے فائدہ مند ہے؟	☐	☐	☐
17. Do you know how to evaluate your curative effect?<br>کیا آپ جانتے ہیں کہ دی جانے والی ادویات سے آپکی بیماری میں فائدہ ہو رہا ہے ؟	☐	☐	☐
18. Do you know how to contact medical staffs when you have a question?<br>کیا آپ جانتے ہیں کہ جب کوئی مشکل پیش آئے تو آپ اپنے معلج (ڈاکٹر) سے کیسے رابطہ کر سکتے ہیں؟	☐	☐	☐

END OF QUESTIONS. THANK YOU سوالات کا اختتام۔ شکریہ
